# Immune Response in a Wild Bird Is Predicted by Oxidative Status, but Does Not Cause Oxidative Stress

**DOI:** 10.1371/journal.pone.0122421

**Published:** 2015-03-27

**Authors:** Dominic L. Cram, Jonathan D. Blount, Jennifer E. York, Andrew J. Young

**Affiliations:** 1 Centre for Ecology & Conservation, College of Life & Environmental Sciences, University of Exeter, Penryn Campus, Cornwall, United Kingdom; 2 Department of Zoology, University of Cambridge, Cambridge, United Kingdom; 3 Department of Zoology and Entomology, University of Pretoria, Pretoria, Gauteng, South Africa; Institute of Zoology, CHINA

## Abstract

The immune system provides vital protection against pathogens, but extensive evidence suggests that mounting immune responses can entail survival and fecundity costs. The physiological mechanisms that underpin these costs remain poorly understood, despite their potentially important role in shaping life-histories. Recent studies involving laboratory models highlight the possibility that oxidative stress could mediate these costs, as immune-activation can increase the production of reactive oxygen species leading to oxidative stress. However, this hypothesis has rarely been tested in free-ranging wild populations, where natural oxidative statuses and compensatory strategies may moderate immune responses and their impacts on oxidative status. Furthermore, the possibility that individuals scale their immune responses according to their oxidative status, conceivably to mitigate such costs, remains virtually unexplored. Here, we experimentally investigate the effects of a phytohaemagglutinin (PHA) immune-challenge on oxidative status in wild male and female white-browed sparrow weavers, *Plocepasser mahali*. We also establish whether baseline oxidative status prior to challenge predicts the scale of the immune responses. Contrary to previous work on captive animals, our findings suggest that PHA-induced immune-activation does not elicit oxidative stress. Compared with controls (n = 25 birds), PHA-injected birds (n = 27 birds) showed no evidence of a differential change in markers of oxidative damage or enzymatic and non-enzymatic antioxidant protection 24 hours after challenge. We did, however, find that the activity of a key antioxidant enzyme (superoxide dismutase, SOD) *prior* to immune-activation predicted the scale of the resulting swelling: birds with stronger initial SOD activity subsequently produced smaller swellings. Our findings (i) suggest that wild birds can mount immune responses without suffering from systemic oxidative stress, and (ii) lend support to biomedical evidence that baseline oxidative status can impact the scale of immune responses; a possibility not yet recognised in ecological studies of immunity.

## Introduction

Immune systems have evolved to minimize the detrimental effects of pathogen infection on host fitness. However, mounting immune responses may entail significant costs, in part because the associated investment may be traded-off against resource allocation to other fitness-related functions [1]. Extensive evidence suggests that mounting a wide variety of immune responses can lead to long-term costs to fecundity and survival [2–4]. Despite their potentially important role in shaping life-histories, our understanding of the molecular mechanisms that mediate such costs remains poorly developed [5].

Traditionally, the costs entailed in mounting immune responses were thought to arise from trade-offs in the allocation of energy [1, 6], yet a recent meta-analysis found only small, recoverable energetic costs following immune activation [5]. More recently, it has been suggested that the long-term costs of an immune response may be mediated by the damaging effects of reactive oxygen species (ROS), which may be more difficult to recover compared to minor energy deficits [5, 7]. ROS are highly reactive molecules produced during aerobic respiration, cell-signalling and immune defence, and can inflict significant oxidative damage to DNA, lipids and proteins [8]. Such harmful effects are usually minimized by the body’s antioxidant system, but when ROS overwhelm antioxidant defences, a state of oxidative stress results [9]. Oxidative stress has been implicated in many of the same long-term impairments highlighted as costs of mounting immune responses, including reproductive dysfunction and accelerated senescence [10, 11]. Immune responses in particular can enhance ROS production, as macrophages and heterophils generate ROS directly in response to foreign antigens, in order to kill pathogens during ‘respiratory burst’ [12, 13]. Inflammation (which commonly occurs during infection or injury) can also elevate ROS generation [14]. Whether the non-specific destructive action of ROS produced during respiratory burst and inflammation can induce systemic oxidative stress, thereby eliciting the documented long-term costs of immune activation, remains unclear.

A recent meta-analysis found that a range of experimental immune activation treatments typically do cause oxidative stress in birds, though there was considerable heterogeneity among studies [7]. However, these studies were almost exclusively conducted in captivity, with studies of wild animals typically involving either transfers to captivity [15, 16], or the use of very young animals without fully developed immune and antioxidant systems [17]. Whether adults in wild populations will reflect or contradict findings in captivity remains virtually unexplored. This is important, as the artificial conditions experienced by captive animals, including unlimited access to food and water, novel diet compositions, limited resource demands and unnatural exposure to stressors all have the potential to impact both oxidative status and the scale and associated costs of an immune response. As such, studies that experimentally challenge the immune systems of wild animals, without transfers to captivity, are now required to advance our understanding of the interactions between immunity and oxidative physiology [5].

If mounting a particular immune response entails an oxidative cost, selection may favour the modulation of the strength of that immune response according to the individual’s baseline oxidative status. Individuals already suffering from oxidative stress might therefore be predicted to mount correspondingly weak immune responses, in order to minimize oxidative damage (provided such benefits outweigh any associated cost of reduced defence against infection). Indeed, evidence from male painted dragon lizards (*Ctenophorus pictus*) suggests that individuals with high levels of superoxide radicals subsequently do mount weaker immune responses following mitogenic immune activation [18]. Alternatively, an individual’s baseline oxidative status could directly impact aspects of an immune response. For example, extensive biomedical evidence supports a role for strong antioxidant defences in reducing the severity of inflammatory immune responses [19, 20]. Baseline oxidative status might therefore play a key role in determining the scale and associated costs of an immune response in a manner not yet widely recognised in ecological studies of immunity.

In this study, we use a phytohaemagglutinin (PHA) immune challenge to experimentally test whether immune activation causes oxidative stress in a wild bird, the white-browed sparrow weaver (*Plocepasser mahali*). PHA is widely used in ecological studies of immunity and is known to induce a complex immunological cascade, including the proliferation of lymphocytes, the recruitment of heterophils and macrophages, and the promotion of a local inflammatory response [21–23]. The immune response to PHA challenge may therefore be expected to elevate ROS production through a number of mechanisms (see above), and evidence from captive birds suggests that it can indeed increase oxidative damage [15].

We sampled the oxidative status of 52 wild birds immediately prior to the administration of a PHA challenge or a saline injection control, and again on recapture from the wild 24 hours later, to address three key aims. First, we investigate whether birds subjected to a PHA challenge differ in oxidative status after 24 hours from saline-injected birds (while controlling for variation in their baseline oxidative status prior to challenge). Second, we investigate whether the size of an individual’s swelling at the site of PHA injection predicts the magnitude of their change in oxidative status. Wing-web swelling is a frequently measured physical response to PHA injection [24], and there is evidence it scales with levels of local infiltration of leukocytes and systemic phagocytic activity [22, 25]. Stronger swelling responses (indicating increased immune cell activity) may therefore be predicted to have greater impacts on oxidative status. Finally, we investigate whether an individual’s baseline oxidative status prior to PHA challenge predicts the strength of their swelling response to that challenge, which might be predicted if individuals mitigate the oxidative costs of immune responses by modulating their response according to their oxidative status.

Oxidative status is a complex, multi-faceted physiological parameter that can only be characterised through multiple markers of antioxidant protection and oxidative damage [26]. We therefore investigate a suite of metrics relevant to oxidative status: circulating levels of a lipid oxidative damage product (malondialdehyde, MDA), intra-cellular enzymatic antioxidant protection in the form of superoxide dismutase (SOD) activity, and circulating non-enzymatic antioxidant protection (Total Antioxidant Capacity (TAC) controlling for the confounding effects of uric acid [27]). Previous studies have shown that these measures of oxidative status are sensitive to a range of experimental immune-challenges [15, 28–30].

## Methods

### Study population and field methods

All protocols have been approved by the University of Pretoria, South Africa, ethics committee, were conducted under permit from SAFRING (license 1444) and Northern Cape Conservation, and conform with the guidelines for the use of animals in research. Permission to conduct fieldwork was provided by the land-owners, Ernest Oppenheimer and Son.

The experiment was conducted using a wild population of 20 cooperatively breeding groups of white-browed sparrow weavers at Tswalu Kalahari Reserve, South Africa (27°16'S, 22°25’E). Data collection took place between November 2011 and February 2012 during the breeding season, but when group members were not provisioning offspring. Breeding stage was confirmed by nest searches every two days. All experimental birds were at least seven months old, and were fitted with a metal ring for identification. Males and females were distinguished by beak colour: males have dark brown beaks while females have paler horn-coloured beaks [31]. Socially dominant birds are the principal breeders [32, 33] and so were excluded from the study to minimise among-individual variance in reproductive status, and avoid negatively impacting reproductive success [2, 34]. Dominance was assigned following weekly behavioural observations and criteria detailed elsewhere [32, 35].

### Experimental design

Birds from 20 cooperative groups were alternately assigned to one of two treatment groups: PHA-challenged (n = 27 birds; 13 males, 14 females) or control-injected (n = 25 birds; 12 males, 13 females). During the evening of Day 1, birds were captured from their roost chambers and blood sampled (see below) for the determination of their baseline levels of oxidative damage and antioxidant protection. Wing-web thickness at the site of injection was measured (see below), and individuals were then injected with either PHA solution or phosphate buffered saline (PBS) and returned to their roost chambers to pass the remainder of the night and freely range the following day. On the subsequent evening (Day 2), the same birds were recaptured 24 hours after their injection (mean ± standard deviation (SD) = 24.02 ± 0.8 hours) and blood sampled to reveal their state after treatment, before being returned once again to their roost chambers (see S1 File for full dataset).

### Captures, blood-sampling and immune challenge

All captures and blood sampling were conducted by one person. Birds were captured individually at night, by flushing them from their individual roost chambers into a custom capture bag. A blood sample (approximately 160 μl) was immediately collected from the brachial vein with a 26g needle (mean ± SD time lag from capture to bleed completion = 188 ± 75 seconds). All blood samples were collected from the left wing and all injections were administered to the right wing, to avoid possible localized effects of blood sampling on wing-web thickness. The thickness of the wing-web (patagium) on the right wing was then measured three times using a pressure sensitive calliper (Model 700–118; Mitutoyo, Japan). These wing-web thickness measures were highly repeatable [36] both pre-injection (F_51,104_ = 90.00, r = 0.967, p < 0.001) and post-injection (F_51,104_ = 242.78, r = 0.988, p < 0.001). The injection was administered as follows: the wing-web was sterilized with ethanol and injected subcutaneously with either a solution of 0.02 mg PHA (L8754; Sigma, UK) in 0.04 ml autoclaved PBS (P4244; Sigma, UK), or a control solution of 0.04 ml PBS, following [37]. The ‘swelling response’ to injection was calculated as the difference between the means of the wing-web thickness measures taken immediately before and 24 hours after injection.

### Blood processing and oxidative status metric determinations

After collection, blood was immediately separated by centrifugation in the field (12,000 × g for 3 minutes, Haematospin 1400; Hawksley Medical and Laboratory Equipment, UK). Erythrocytes drawn from the cellular phase of the separated whole blood were immediately lysed by combining them with four times their volume of ice-cold distilled water, mixing this solution, and placing it on ice for 5 minutes. This solution was then centrifuged for 3 minutes (12,000 × g) and the supernatant (erythrocyte lysate) drawn off. Plasma from the separated whole blood (for the determination of MDA, TAC and uric acid levels) and lysed erythrocytes (for the determination of SOD activities) were stored on ice until they could be transferred to liquid nitrogen on return to base camp (mean ± SD time lag from sampling to storage on liquid nitrogen: 149 ± 66 minutes). Samples were transported from the field site to the UK on dry ice where they were stored at -80°C until analysis within 12 months.

#### Oxidative damage to lipids (MDA)

Plasma concentrations of malondialdehyde (MDA) were determined by high performance liquid chromatography, following [38]. A subset of plasma samples run in duplicate showed high repeatability (F_66,67_ = 15.92, r = 0.88, p < 0.001).

#### Enzymatic antioxidant protection (SOD)

The SOD activity in erythrocyte lysate was determined using a colorimetric assay (Cayman Chemicals, USA) and a spectrophotometer (Spectramax M2; Molecular Devices, USA). One unit is defined as the amount of enzyme needed to exhibit 50% dismutation of the superoxide radical; enzyme activities are reported as units/ml. A subset of samples were analysed in duplicate on separate plates, which confirmed that SOD activities were highly repeatable between plates (F_37,38_ = 6.07, r = 0.72, p < 0.001).

#### Total Antioxidant Capacity (TAC)

We estimated non-enzymatic ‘Total Antioxidant Capacity’ (TAC) by measuring the capacity of a plasma sample to quench a standardised free radical challenge. Plasma TAC was determined using a colorimetric assay kit (Cayman Chemicals, USA) and spectrophotometer (Spectramax M2; Molecular Devices, USA). Plasma TAC values are expressed as Trolox-equivalent antioxidant concentrations. A subset of plasma samples were run in duplicate on separate plates, which confirmed that TAC values were highly repeatable between plates (F_41,42_ = 8.20, r = 0.78, p < 0.001).

It has recently been highlighted that, in some avian species, a significant portion of the antioxidant activity measured by the TAC assay may be due to the antioxidant effects of uric acid, the primary nitrogen waste product in birds [39, 40]. The importance of uric acid as an antioxidant *in vivo* remains unclear [40, 41], leaving avian plasma TAC values potentially confounded by the ‘incidental’ antioxidant activity of uric acid [27]. Indeed, in our study plasma uric acid concentrations significantly predicted plasma TAC (linear mixed model with bird identity as a random factor, χ^2^_1_ = 37.89, p < 0.001, n = 104 samples; model estimate: 0.30 ± 0.045, conditional R^2^ = 0.40 [42]). We therefore calculated the residuals from a linear model with TAC as the response term and uric acid concentration as the sole predictor, to yield a measure of plasma antioxidant capacity excluding that arising from uric acid (hereafter termed ‘residual TAC’). See [27, 43] for further details.

#### Uric acid

Plasma concentrations of uric acid were determined using a fluorescence assay kit (Cayman Chemical, USA) and spectrophotometer (Spectramax M2; Molecular Devices, USA). A subset of plasma samples were run in duplicate on separate plates, which confirmed that uric acid concentrations were highly repeatable between plates (F_39,40_ = 8.35, r = 0.79, p < 0.001).

### Statistical analysis

Statistical analyses were carried out using R 2.15.1 [44], using a step-wise model simplification approach [45]. Initially all fixed terms of interest were fitted (see below for details), followed by the stepwise deletion of terms whose removal resulted in a non-significant change in deviance (using a likelihood-ratio test for model comparison), until the minimal adequate model (MAM) was obtained, in which only significant terms remained. Dropped terms were then added back in to the MAM to confirm their non-significance and were retained in the MAM when found to be significant in this context. The homoscedasticity and normality of residuals were inspected and, where necessary, response terms were transformed to satisfy these criteria. Sparrow weaver social group was fitted as a random term in all models, to control for sampling of multiple individuals within social groups (52 individuals were sampled across 20 social groups).

#### 1) Does PHA challenge affect an individual’s oxidative status?

Four linear mixed models were used to assess the effect of treatment (PHA challenge vs. PBS injection) on an individual’s post-challenge levels of each of the three key metrics of oxidative status (MDA concentration, SOD activity, TAC residuals) as well as uric acid concentration. The post-challenge level of a given oxidative status measure was fitted as the response term and the pre-challenge level of the same measure was fitted as a covariate predictor alongside treatment. This approach is more statistically powerful than modelling the effect of treatment on the *change* in a given oxidative status measure and can also account for the effects of chance biases in the treatment groups in the pre-challenge levels of the focal oxidative status metric [45]. Bird sex was also included as a factorial predictor, as well as all two-way interactions among terms.

Post-treatment plasma concentrations of MDA and uric acid were log-transformed, and post-treatment SOD activity was square-root-transformed. Sample sizes differ slightly among the analyses (see results) as all four physiological metrics could be quantified for the vast majority of birds both pre- and post-challenge, but not for all (47 of 52).

#### 2) Does an individual’s swelling response to PHA challenge predict the magnitude of their associated change in oxidative status?

A similar set of four mixed models were used to examine whether the magnitude of the swelling response to PHA challenge predicted an individual’s post-challenge oxidative status metrics. The pre-challenge level of the focal oxidative status measure was fitted as a covariate predictor, along with the swelling response (mm), sex, and all two-way interactions among terms. Post-treatment plasma concentrations of MDA and uric acid were log-transformed, and post-treatment SOD activity was square-root-transformed.

#### 3) Does an individual’s pre-challenge oxidative status predict the strength of their swelling response to PHA challenge?

Finally, a single linear mixed model was used to test whether pre-challenge oxidative status metrics predicted the magnitude of the bird’s swelling following PHA challenge. Swelling response (mm) was set as the response term and the pre-challenge levels of the three focal oxidative status metrics as well as uric acid concentration and pre-challenge body mass were fitted as covariate predictors. Sex was fitted as a factor. To normalise model residuals, swelling response was square-root transformed and SOD activity was log-transformed.

## Results

### 1) Does PHA challenge affect an individual’s oxidative status?

The birds allocated to the two treatment groups showed no significant differences in their pre-challenge levels of any of the three key oxidative status metrics or uric acid (all t < 0.61, p > 0.55). As expected, the birds injected with PHA showed significantly larger wing-web swelling responses after 24 hours than those injected with PBS (mean change in wing-web thickness ± standard error (S.E.): PHA = + 0.42 ± 0.03 mm, PBS = + 0.07 ± 0.01 mm, linear mixed model: χ^2^_1_ = 73.49, p < 0.001, n = 52 birds). Within PHA-challenged birds, we found no effect of body mass on swelling size (General Linear Mixed Model with social group as the random factor: χ^2^_1_ = 0.50, p = 0.48, n = 27 birds); our results support previous evidence that there is no mass-dependent effect of PHA-challenge [15, 46]. There was also no significant effect of sex on the swelling response, either as a single factor or in an interaction between sex and treatment (both χ^2^_1_ < 0.67, p > 0.41). Moreover, as outlined below, we found no significant effect of treatment (PHA challenge versus PBS injection) on any of our three focal metrics of oxidative status.

**Fig 1:**
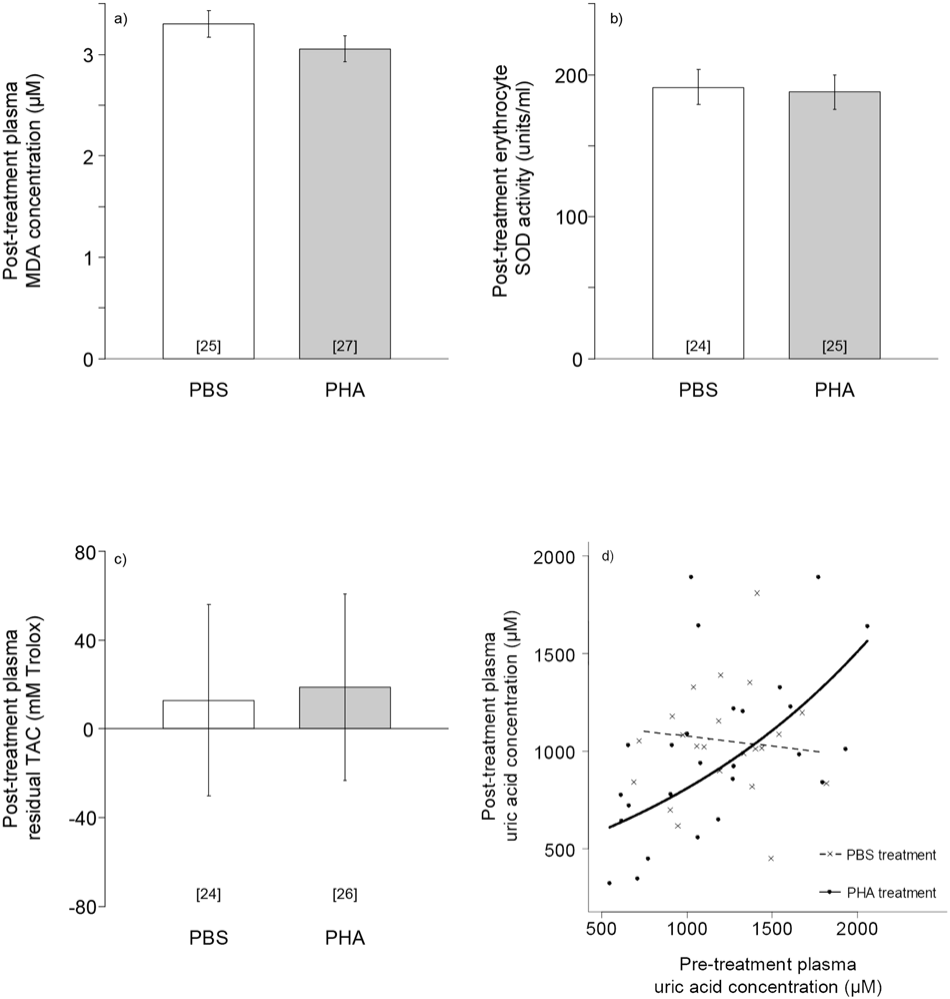
The effect of PHA immune activation on measures of oxidative status. Relative to PBS-injected controls, PHA challenge had no significant effect on: **(a)** plasma concentrations of maldondialdehyde (MDA); (**b)** the activity of the antioxidant enzyme superoxide dismutase in erythrocytes (SOD); **(c)** plasma total antioxidant capacity excluding the effects of uric acid (residual TAC). (**d)** Post-injection plasma uric acid concentrations *were* predicted by a signficiant interaction between treatment and baseline plasma uric acid concentrations. In (a), (b) and (c), bars show model predictions ± S.E. from linear mixed models with treatment as the only predictor. Numbers in parentheses denote sample sizes. In (d), circles are PHA birds, crosses are PBS control birds. Lines represent model predictions for PHA (solid line) and PBS-injected birds (dashed line) from a linear mixed model containing the interaction of treatment and pre-treatment uric acid levels.

There was no significant effect of PHA challenge on post-treatment plasma MDA concentrations (Fig. 1a, χ^2^_1_ = 2.07, p = 0.15, n = 52 birds), nor were there significant effects of sex or pre-treatment MDA levels (both χ^2^_1_ < 1.39, p > 0.24). Similarly, there was no significant effect of PHA challenge on post-treatment erythrocyte SOD activity (Fig. 1b, χ^2^_1_ = 0.39, p = 0.53, n = 49 birds) while controlling for a significant interaction between sex and pre-treatment SOD activity (χ^2^_1_ = 7.70, p = 0.006). This interaction arose because, regardless of treatment, pre- and post-treatment SOD activities were strongly positively correlated in females (slope ± S.E.: +1.02 ± 0.07), but post-treatment SOD was typically lower than pre-treatment SOD in males (slope ± S.E.: 0.83 ± 0.05). There was also no significant effect of treatment on plasma residual TAC (Fig. 1c, χ^2^_1_ = 0.03, p = 0.87, n = 50 birds), nor were there significant effects of pre-treatment residual TAC or sex (both χ^2^_1_ < 2.25, p > 0.13). There was, however, an effect of treatment on post-treatment plasma uric acid concentrations, as evidenced by a significant interaction between treatment and pre-treatment uric acid concentration (Fig. 1d, χ^2^_1_ = 5.93, p = 0.01, n = 50 birds), while controlling for a significant interaction between sex and pre-treatment uric acid concentration (χ^2^_1_ = 7.26, p = 0.007). Treatment with PHA (relative to PBS) had a negative effect on post-treatment uric acid levels in birds with low pre-treatment uric acid levels, but a positive effect in those with high levels of uric acid before treatment (Fig. 1d). Within the PBS-treated birds, pre-treatment uric acid levels did not affect those post-treatment.

### 2) Does an individual’s swelling response to PHA challenge predict the magnitude of their associated change in oxidative status?

Among PHA challenged birds, the scale of an individual’s swelling response to PHA challenge did not significantly predict any aspect of its post-challenge oxidative status: post-treatment plasma MDA concentration (χ^2^_1_ = 1.05, p = 0.31, n = 27 birds; while controlling for a positive effect of pre-treatment MDA concentration: χ^2^_1_ = 5.20, p = 0.023); post-treatment erythrocyte SOD activity (χ^2^_1_ = 0.33, p = 0.57, n = 25 birds; while controlling for a significant interaction between pre-treatment SOD activity and sex: χ^2^_1_ = 5.40, p = 0.020); post-treatment residual TAC (χ^2^_1_ = 0.14, p = 0.71, n = 26 birds); post-treatment uric acid concentration (χ^2^_1_ = 0.65, p = 0.41, n = 26 birds; while controlling for a significant interaction between pre-treatment uric acid concentration and sex: χ^2^_1_ = 6.33, p = 0.012).

### 3) Does an individual’s pre-challenge oxidative status predict the strength of their PHA-induced swelling?

**Fig 2:**
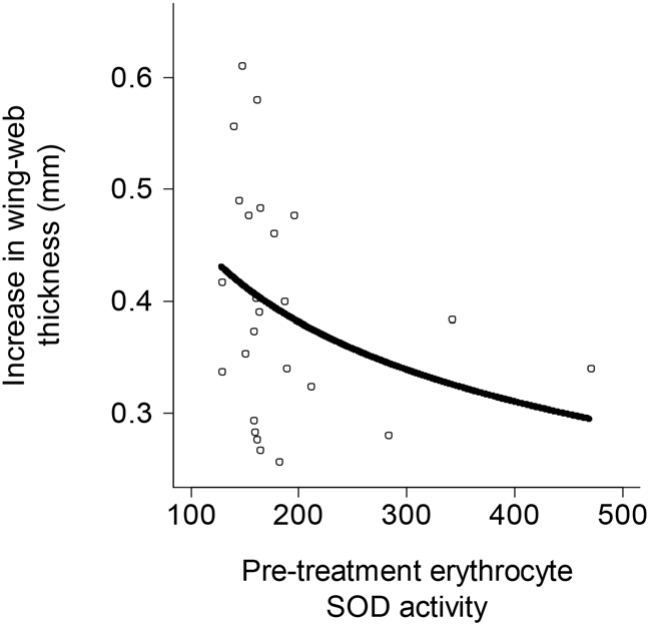
The association between pre-treatment SOD activity and subsequent swelling response at the PHA injection site. Birds with higher pre-treatment erythrocyte SOD activities subsequently exhibited smaller swelling following PHA challenge (n = 25 birds). The solid line depicts model predictions from a GLMM with swelling as the response and pre-treatment SOD activity as the only predictor. The three birds with the highest pre-treatment SOD activities also had correspondingly high post-treatment SOD activities, and we reconfirmed these high pre- and post-treatment values by re-running the samples in duplicate on separate plates. These high activities do not therefore appear to be artifacts of laboratory procedures.

Pre-treatment erythrocyte SOD activity significantly predicted the size of the subsequent swelling response to PHA challenge (Fig. 2, χ^2^_1_ = 5.94, p = 0.015, n = 26 birds); those birds with higher pre-treatment SOD activities exhibited smaller swellings after PHA challenge. The swelling response was not predicted by pre-challenge body mass or plasma concentrations of MDA, uric acid or TAC residuals (χ^2^_1_ < 0.76, p > 0.38), or by sex (χ^2^_1_ = 1.33, p = 0.25).

## Discussion

To our knowledge this is the first experimental study to investigate the effects of PHA-mediated immune activation on oxidative status in a wild, free-ranging adult bird without transfer to captivity. Our results suggest that PHA-induced immune activation does not cause changes in key circulating metrics of oxidative status, including oxidative damage to lipids and measures of both enzymatic and non-enzymatic antioxidant protection. Immune activation did affect plasma levels of uric acid, though not in a straightforward way: PHA-challenge (relative to PBS) had a negative effect on post-treatment uric acid levels in birds with low pre-treatment uric acid levels, but a positive effect on post-treatment uric acid levels in those with high pre-treatment uric acid levels. Within PHA challenged birds, the size of the swelling at the injection site (a proxy for the strength of the immune response [22, 23, 25]) did not predict the associated changes in any of the oxidative status metrics. Finally, pre-treatment erythrocyte SOD activity predicted the strength of the swelling response following PHA challenge: birds with higher initial SOD protection subsequently produced a smaller swelling after PHA challenge. Together, these findings suggest that immune activation by PHA challenge has no net effect on circulating markers of oxidative status in the wild, but that the scale of the response to such a challenge may be modulated according to baseline oxidative status.

A subcutaneous PHA challenge of the type administered here has been shown to trigger the infiltration and proliferation of a number of immune cell types (heterophils, macrophages, basophils and thrombocytes [22]) and a localised inflammatory response [23] after 24 hours. Given the documented effects of the heterophil/neutrophil respiratory burst on oxidative damage to host cells and tissues *in vitro* [47, 48], the immunological responses to PHA challenge are thought to induce oxidative stress either locally or systemically [17]. While previous work suggests that PHA challenge can indeed negatively impact oxidative status reflected in circulating markers [15, 17] after 24 hours, our findings on the same timescale reveal no clear impact of PHA challenge on circulating markers of oxidative damage or antioxidant protection, suggesting that any cost reflected in these currencies may be minimal in wild, free-ranging adults. Consistent with this finding, our results also indicate that individuals that exhibited larger swellings after PHA challenge did not experience larger changes in oxidative status. While we examined a suite of oxidative stress markers, PHA-induced immune activation could conceivably have impacted other markers not investigated here (e.g. DNA or protein damage products), or caused oxidative damage to tissues in a manner unrelated to circulating markers, potentially local to the site of the challenge. Indeed, immune challenge *can* impact tissue levels of carotenoids [49], and tissue-specific changes in oxidative status may not always be reflected in circulating markers [50]. Alternatively, the net impact of PHA-challenge on oxidative status may be minimized in the wild by compensatory behavioural or physiological adjustments. First, animals may reduce investment in costly activities to potentially decrease associated ROS production. Indeed, evidence from our study population suggests that dominant males markedly reduce their dawn song output following a PHA challenge identical to that used in the current study [51]. Second, increased synthesis or intake of antioxidants could offer protection even where ROS production is elevated (e.g. ‘self-medication’ with food rich in antioxidants [52]). Strategic reduction of ROS production and/or additional antioxidant protection may therefore allow individuals to avoid the oxidative stress costs of immune activation. Such strategies may explain evidence that infection and the associated immune response *can* promote oxidative stress, but only during reproduction [53, 54], when the above behavioural and physiological compensatory mechanisms may be difficult to achieve without risking the loss of the offspring.

PHA-challenge negatively affected levels of uric acid, but only in those birds that had low initial circulating uric acid concentrations. Uric acid’s contribution to *in vitro* plasma measures of antioxidant activity has only recently been highlighted in ecological studies [27, 40, 55]. However, *in vivo* evidence of uric acid’s antioxidant activity is mixed, with uric acid frequently *promoting* oxidative stress [56] or other serious diseases [57, 58]. In birds, uric acid is the primary form in which nitrogen is excreted, and is produced during amino acid breakdown, so reduced circulating uric acid levels may simply reflect a reduction in foraging in response to immune challenge (e.g. ‘sickness behaviours’ commonly reported following immune activation [34, 59]). Alternatively, reduced circulating uric acid could reflect an increase in ROS production interacting with circulating uric acid, if ROS convert uric acid to its oxidation product allantoin [60]. Future studies might therefore usefully investigate the impacts of putative drivers of oxidative status on the ratios of uric acid to allantoin, with a view to providing greater insight in to the biological significance of uric acid as an antioxidant [61].

Our results reveal that the magnitude of the swelling response to PHA challenge was predicted by the pre-challenge activity of a key antioxidant enzyme (SOD); birds with *higher* initial SOD protection subsequently produced *smaller* swellings in response to PHA challenge. As SOD activity is frequently up-regulated in response to oxidative stress (see [62] and references therein), this finding could conceivably be consistent with the prediction that individuals with poor oxidative statuses (who may therefore express SOD at higher rates) should limit their immune responses in order to mitigate the associated oxidative costs [18]. Indeed, such strategic modulation could conceivably account for the lack of any clear effect of immune challenge on overall oxidative status. Alternatively, this finding could reflect direct mitigating effects of the antioxidant system on the inflammatory component of immune responses [63, 64]. PHA-mediated swelling encompasses increased local blood supply and cellular infiltration in response to the foreign antigen and its damage to local tissues [21]. While such inflammatory responses represent an essential adaptive mechanism for the protection of tissue integrity, oxidative stress can promote excessive inflammatory responses [63], which can lead to systemic, long-lasting loss of homeostasis (e.g. neurodegenerative disease, heart failure and cancer, reviewed in [65]). Extensive bio-medical evidence suggests that antioxidants can mitigate the negative effects of inflammation [19, 20, 66]. Indeed, SOD itself can have a strong anti-inflammatory action [64], which could arise through at least two pathways. First, SOD can reduce the expression of pro-inflammatory cytokines by blocking their redox-sensitive transcription factors [67]. Second, elevated SOD activity removes superoxide ions before they can locally recruit neutrophils to promote further inflammation [68, 69]. The central role played by SOD in controlling inflammation and its harmful effects is thought to explain why an otherwise lethal influenza infection can be fully alleviated by injection or over-expression of SOD in captive mice [70, 71]. Our results therefore highlight the possibility that SOD plays a similar role in regulating inflammation in natural, wild populations. Further research is now needed to investigate whether any SOD-associated tempering of the inflammatory response in the wild yields fitness benefits, for example by permitting faster recovery following immune activation.

In conclusion, our findings indicate that PHA-induced immune activation does not impact key circulating metrics of oxidative status in a wild bird. While a complex effect of the challenge on circulating uric acid concentrations was detected, the biological significance of the antioxidant properties of uric acid remains unclear. This lack of an apparent oxidative stress cost may arise because the immune response to PHA challenge has little impact on the balance of ROS production and antioxidant protection, or because individuals make compensatory behavioural or physiological adjustments that mitigate the net impact on oxidative status. Either way, our findings do not support the hypothesis that the costs of mounting immune responses are mediated by the concomitant induction of oxidative stress. Further experimental studies on wild populations might now usefully investigate the effects on oxidative status of immunological responses to stronger or more systemic challenges. Our findings also concord with evidence from the biomedical literature suggesting that baseline antioxidant protection may play a key role in modulating the inflammatory response to immune challenge, a possibility not yet widely recognised in ecological studies of immunity. This study highlights the importance of investigating interactions between oxidative physiology and immunity in wild populations, where natural oxidative statuses and the scope to mount natural compensatory responses may impact the net costs experienced when wild animals mount immune responses.

## Supporting Information

S1 File Data used in the analyses of “Immune response in a wild bird is predicted by oxidative status, but does not cause oxidative stress”. (TXT)
